# Stepwise metabolic engineering of *Escherichia coli* to produce triacylglycerol rich in medium-chain fatty acids

**DOI:** 10.1186/s13068-018-1177-x

**Published:** 2018-06-25

**Authors:** Lin Xu, Lian Wang, Xue-Rong Zhou, Wen-Chao Chen, Surinder Singh, Zhe Hu, Feng-Hong Huang, Xia Wan

**Affiliations:** 1 Oil Crops Research Institute of the Chinese Academy of Agricultural Sciences, Wuhan, 430062 People’s Republic of China; 2 CSIRO Agriculture & Food, Canberra, 2601 Australia; 3 Key Laboratory of Biology and Genetic Improvement of Oil Crops, Ministry of Agriculture, Wuhan, 430062 People’s Republic of China; 4 Oil Crops and Lipids Process Technology National & Local Joint Engineering Laboratory, Wuhan, 430062 People’s Republic of China; 5 Hubei Key Laboratory of Lipid Chemistry and Nutrition, Wuhan, 430062 People’s Republic of China; 6 State Key Laboratory of Agricultural Microbiology, Huazhong Agricultural University, Wuhan, 430070 People’s Republic of China

**Keywords:** *Escherichia coli*, Triacylglycerol, Medium-chain fatty acid, Acyltransferase, Acyl-ACP thioesterase, Lipid droplet

## Abstract

**Background:**

Triacylglycerols (TAGs) rich in medium-chain fatty acids (MCFAs, C10–14 fatty acids) are valuable feedstocks for biofuels and chemicals. Natural sources of TAGs rich in MCFAs are restricted to a limited number of plant species, which are unsuitable for mass agronomic production. Instead, the modification of seed or non-seed tissue oils to increase MCFA content has been investigated. In addition, microbial oils are considered as promising sustainable feedstocks for providing TAGs, although little has been done to tailor the fatty acids in microbial TAGs.

**Results:**

Here, we first assessed various wax synthase/acyl-coenzyme A:diacylglycerol acyltransferases, phosphatidic acid phosphatases, acyl-CoA synthetases as well as putative fatty acid metabolism regulators for producing high levels of TAGs in *Escherichia coli*. Activation of endogenous free fatty acids with tailored chain length via overexpression of the castor thioesterase RcFatB and the subsequent incorporation of such fatty acids into glycerol backbones shifted the TAG profile in the desired way. Metabolic and nutrient optimization of the engineered bacterial cells resulted in greatly elevated TAG levels (399.4 mg/L) with 43.8% MCFAs, representing the highest TAG levels in *E. coli* under shake flask conditions. Engineered cells were observed to contain membrane-bound yet robust lipid droplets.

**Conclusions:**

We introduced a complete Kennedy pathway into non-oleaginous *E*. *coli* towards developing a bacterial platform for the sustainable production of TAGs rich in MCFAs. Strategies reported here illustrate the possibility of prokaryotic cell factories for the efficient production of TAGs rich in MCFAs.

**Electronic supplementary material:**

The online version of this article (10.1186/s13068-018-1177-x) contains supplementary material, which is available to authorized users.

## Background

Worldwide, demand for oil-based products used in the energy industry has increased over recent decades leading to concerns for both the supply and the carbon footprint on the environment. Renewable plant oils, non-seed oils, and microbial oils (or single-cell oils, SCOs) are expected to play vital roles in providing a sustainable source of oils [1, 2]. Triacylglycerols (TAGs) rich in medium-chain fatty acids (MCFAs), including capric acid (C10:0), lauric acid (C12:0), and myristic acid (C14:0), are of particular interest due to their lower freezing point and higher carbon conversion yield. Such oils are important constituents of certain oleochemicals and jet fuels [3–5]. However, the end products of wild-type plant and bacterial fatty acid synthetase activities are mainly C16 and C18 fatty acids. Meanwhile, natural sources of oils rich in MCFAs are restricted to a limited number of plant species, such as *Cinnamomum camphora*, *Cuphea* sp., *Cocos nucifera,* and *Umbellularia california*, which are unsuitable for mass agronomic production [6].

Several studies have investigated the modification of seed oils to increase MCFA content. Co-expression of MCFA-specific *CpuDGAT1*, *CvFatB1,* and *CvLPAT2* resulted in 25 mol% of C10 fatty acid in *Camelina* seed oils with modest effects on seed oil content and plant growth [7]. In addition, the accumulation of MCFAs in *Nicotiana benthamiana* leaf oils has been recently achieved by introduction of MCFA-specific *EgDGAT* along with *CcFatB* or *UcFatB* [8]. Although microbes including *Escherichia coli* and *Yarrowia lipolitica* have been engineered to produce medium-chain free fatty acids, fatty alcohols, fatty acid ethyl esters (FAEEs), or fatty alkanes with varying outcomes [9–11], the accumulation of MCFAs in the form of TAGs is rare in microorganisms, especially prokaryotic microorganisms.

Before achieving accumulation of TAGs rich in MCFAs, TAGs first needs to be synthesized to a high level. The bacterial Kennedy pathway for TAG biosynthesis involves the sequential esterification of glycerol-3-phosphate to produce a central molecular phosphatidic acid (PA) [12]. PA can be dephosphorylated by phosphatidic acid phosphatase (PAP) to produce diacylglycerol (DAG). DAG is subsequently catalyzed into TAG by wax ester synthase/acyl-coenzyme A:diacylglycerol acyltransferase (WS/DGAT). This enzyme is the rate-limiting enzyme, because it leads to the last step of TAG formation. WS/DGATs are unique in oleaginous microorganisms and a few oil crops [13, 14]. Unlike DGAT1 or DGAT2 from eukaryotic algae, plants, and animals, WS/DGAT is the only class of DGAT responsible for TAG formation in bacteria [15]. Among the reported WS/DGATs, AtfA from *Acinetobacter baylyi* has been evaluated as the best candidate for TAG production in *E*. *coli* [16]. Soon after this report, Atf8 from *Rhodococcus jostii* RHA1 was demonstrated to efficiently facilitate TAG accumulation [17]. In addition, a thermophilic tDGAT from *Thermomonospora curvata* was shown to yield over 30% of TAGs to neutral lipids in 30 min in *E*. *coli* [18]. It would be interesting to compare the abilities of these newly isolated WS/DGATs to atfA.

In contrast to WS/DGATs, only a few bacterial PAPs have been characterized for TAG synthesis [19]. Heterologous expression of PAP encoding genes from oleaginous *Streptomyces coelicolor (lppα* or *lppβ)*, or from *R*. *jostii* RHA1 (*ro00075*) has resulted in a two-to-sixfold increase in DAG content in *E. coli* [20, 21]. Furthermore, the combination of LPPα/β along with WS/DGAT Sco0958 leads to TAG accumulation in *E. coli* BL21(DE3)*∆dgkA∆fadE*. Both LPPα and LPPβ are considered to be dedicated PAPs involved in generating DAG for TAG biosynthesis, although LPPβ has a higher PAP activity [21]. The PAP encoding gene *pgpB* is also present in *E. coli*, but its activity is generally low [22]; however, PgpB has been used for enhancement of TAG production [16].

Acyl-CoA synthetase, responsible for esterification of free fatty acids (FFAs) to acyl-CoA, is crucial for reconstruction of the acyl-CoA-dependent TAG synthesis pathway in *E. coli*. There is only a single acyl-CoA synthetase (FadD) with broad substrate specificity identified in *E. coli* [23]. Overexpression of *fadD* enhances the TAG content in engineered *E. coli* [16, 24]. By contrast, numerous acyl-CoA synthetase isoforms are characterized from TAG-producing plants, yeasts, algae, or bacteria [15, 25, 26]. In general, mitochondrial-located acyl-CoA synthetase isoforms activate fatty acids for β-oxidation, while endoplasmic reticulum or plastid-localized isoforms contribute to TAG or PL synthesis in eukaryotic cells. However, whether multiple acyl-CoA synthetases exist and which isoforms of these may be responsible for TAG synthesis in oleaginous bacteria have not been determined yet [15].

The central fatty acid metabolism regulator FadR has been well studied and employed in the previous experiments to increase total fatty acids (TFAs), TAGs, or hydroxy fatty acids in *E. coli* [16, 27]. However, it has also been reported that FadR does not increase the TAG content in *E. coli* containing 4.5% TAGs (w/w CDW) [28]. Although FadR has dual roles for both stimulating fatty acid synthesis and inhibiting fatty acid degradation, it represses *fadD* transcription, and thus impairs recycling of FFA for acyl-CoA-dependent TAG synthesis [29].

Enhancement of MCFA flux is aimed at producing TAGs rich in MCFAs. Since both plants and bacteria use a type II FAS system to synthesize fatty acids, many plant MCFA-specific acyl-ACP thioesterases (TEs) have been successfully applied in *E. coli* for generating high levels of MCFAs [30, 31]. In our recent work, CnFatB3 from *Cocos nucifera*, CcFatB1 from *Cinnamomum camphora,* or CpFatB2 from *Cuphea pulcherrima* have been shown to greatly enhance the MCFA titer in *E. coli* BL21(DE3) [32]. In addition, ChFatB2 from *Cuphea hookeriana* leads to high levels of MCFA accumulation in canola but not in *Camelina* seeds [33, 34]. Whether ChFatB2 can release MCFAs in *E. coli* has not been tested yet. In addition, bacterial TE LpFat or CtFat does not enhance the MCFA titer, although the MCFA percentage was indeed increased in our previous experiments [32]. Among several tested bacterial TEs from the oleaginous bacterium *A*. *babylyi*, only AcTesA’ led to the accumulation of C14:0 in *E*. *coli*, reaching 7.4 µg/mg CDW [35].

**Fig. 1:**
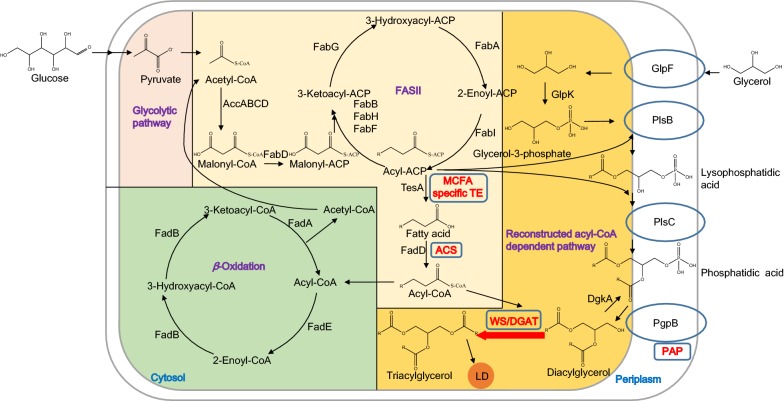
Engineering of *E*. *coli* cells for the production of TAG rich in MCFA. Glycolytic pathway, fatty acid biosynthesis II pathway (FASII), fatty acid degradation pathway (*β*-oxidation), and the reconstructed acyl-CoA-dependent Kennedy pathway are shown in different background colours. Heterologous proteins are highlighted in red. *LD* lipid droplet, *PAP* phosphatidic acid phosphatase, *MCFAs* medium-chain fatty acids, *TE* acyl-ACP thioesterase, *ACS* acyl-CoA synthetase, *WS/DGAT* wax ester synthase/acyl-Coenzyme A:diacylglycerol acyltransferase, *AccABCD* acetyl-CoA carboxyltransferase, *FabA* 3-hydroxydecanoyl-ACP dehydrase, *FabB* 3-ketoacyl-ACP synthase I, *FabD* malonyl-CoA-ACP transacylase, *FabF* 3-ketoacyl-ACP synthase II, *FabG* 3-oxo-acyl-ACP reductase, *FabH* 3-ketoacyl-ACP synthase III, *FabI* enoyl-ACP reductase, *TesA* acyl-CoA thioesterase I, *FadA* 3-ketoacyl-CoA thiolase, *FadB* dodecenoyl-CoA delta-isomerase, *FadD* fatty acyl-CoA synthetase, *FadE* acyl-CoA dehydrogenase, *GlpF* glycerol MIP channel, *GlpK* glycerol kinase, *PlsB* glycerol-3-phosphate acyltransferase, *PlsC* 1-acylglycerol-3-phosphate *O*-acyltransferase, *PgpB* phosphatidylglycerophosphatase B, *DgkA* diacylglycerol kinase

In this study, we focused on the metabolic engineering of *E. coli* aiming to build a sustainable cell platform for the production of TAGs with tailored fatty acids. A Kennedy pathway was reconstructed in *E*. *coli* for the accumulation of high levels of the regular TAGs (Fig. 1). MCFA flux was enhanced by a specific TE and redirected into TAGs. Nutritional optimization further improved the production of TAG rich in MCFAs by 63%, reaching its highest level of 399.4 mg/L in shake flasks. In addition, lipid droplets (LDs) with a robust structure were observed in engineered *E. coli*, suggesting that other value-added lipids may be produced and then stored in the form of prokaryotic LDs with higher stability and less toxicity to cells.

## Results and discussion

### The combination of tDGAT and RoPAP resulted in significant increase inTAGs in *E. coli*

**Fig. 2:**
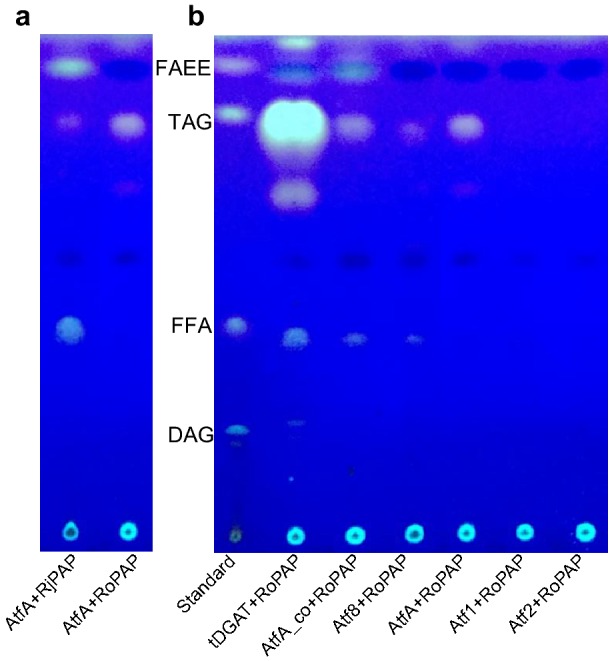
Evaluation of bacterial WS/DGAT and PAP combinations for the production of TAGs in *E*. *coli*. AtfA and RoPAP/RjPAP combinations (**a**) and six different WS/DGATs and RoPAP combinations (**b**). Cells were cultured in ZYP-5052 auto-induction medium at 37 °C with shaking at 200 rpm for 48 h. Thin-layer chromatography (TLC) was carried out on lipids extracted from 10 mg of dried cells. tDGAT: WS/DGAT from *T. curvata*; AtfA: WS/DGAT from *A. baylyi* ADP1; AtfA_co: AtfA codon-optimized for *E. coli*; Atf8: WS/DGAT from *R. jostii* RHA1; Atf1/Atf2: WS/DGATs from *R. opacus* PD630. RoPAP: PAP from *R. opacus* PD630, RjPAP: PAP from *R. jostii* RHA1

WS/DGAT and PAP are the two essential enzymes for reconstruction of the Kennedy pathway in *E. coli*. The expression of AtfA along with RoPAP resulted in an obviously increased TAG, while the expression of AtfA and RjPAP only generated a low amount of TAGs but a higher amount of FAEE and FFA (Fig. 2a). This was consistent with the previous observations that *RjPAP* was not significantly expressed when TAG accumulated in *R. jostii* RHA1 [17]. We predicted that *RjPAP* expression might change the fatty acid composition of the generated DAG, and thus decreased the affinity of AtfA to DAG. Meanwhile, the available cellular acyl-CoA and ethanol could also be catalyzed by wax ester synthase activity of AtfA to form FAEE. Therefore, we then assessed the ability of newly discovered WS/DGAT and RoPAP combinations for TAG production. TLC results showed that the combination of tDGAT and RoPAP produced the most abundant TAG, followed by the combinations AtfA and RoPAP, AtfA_co (the codon-optimized AtfA for *E*. *coli*) and RoPAP, and Atf8 and RoPAP (Fig. 2b). However, Atf1 or Atf2 combined with RoPAP did not yield detectable TAG, although both Atf1 and Atf2 were responsible for TAG formation in *R. opacus* PD630, while Atf2 exhibited higher DGAT activity than Atf1 [36, 37]. Unlike AtfA or Sco0958 (WS/DGAT from *S*. *coelicolor*), overexpression of a solo tDGAT was enough to accumulate considerable TAGs with a threefold increase in monounsaturated fatty acids in *E. coli* C41(DE3) [18, 21, 24]. Combined with the data here, tDGAT was highly active in *E. coli,* and it has the potential for use in the production of functional lipids.

The protein sequences of all tested WS/DGATs contained the catalytic motif HHxxxDG and two other conserved motifs, as shown in Additional file 1: Figure S1. The predicted three-dimensional structure of tDGAT revealed that it had a longer helical linker between the N- and C-terminal domains. In addition, it has been predicted as the most hydrophobic protein among existing representative WS/DGATs, which led to relatively difficult purification [18].

The results here also indicated that DAG availability was crucial for TAG synthesis and that RoPAP contributed to circumvent this bottleneck. Both RoPAP and RjPAP had six predicted transmembrane helices (http://www.cbs.dtu.dk/services/TMHMM/). Sequence alignment of RoPAP and RjPAP showed two conserved domains PSGH and RxxxxHxxxD, which have also been found in LPPα and LPPβ [21]. In addition, the conserved KxxxxxxRP was present in both RoPAP and RjPAP, but absent in PgpB (Additional file 2: Figure S2A). Expression of a solo *RoPAP* or *RjPAP* led to slightly increased TFA content by 14 and 13%, respectively, and the fatty acid profile did not change in either case (Additional file 2: Figure S2B, C).

We also noticed that TAG spots from *E. coli* cells had much stronger signals when staining with primuline over iodine (Additional file 3: Figure S3). In addition, FFA and DAG were more easily observed by staining with primuline (Additional file 3: Figure S3). Therefore, primuline was used for lipid staining in the following tests.

### Acyl-CoA synthetase RofadD1 from *R. opaque* PD630 enhanced TAG production

**Fig. 3:**
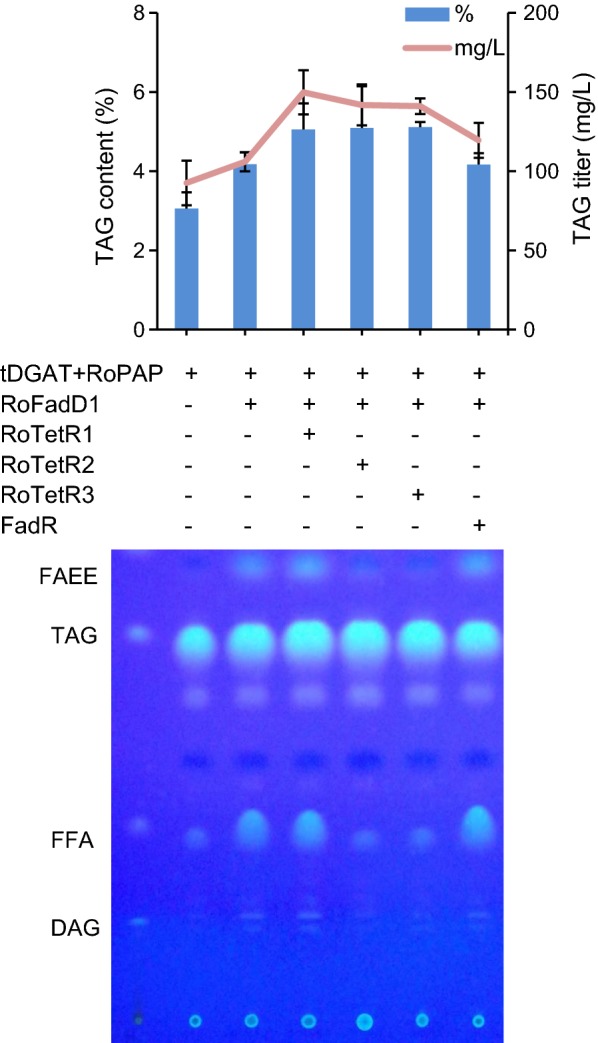
Coexpression of *RoFadD1* and *RoTetRs* along with *tDGAT* and *RoPAP* further increased TAG synthesis. Cells were cultured in ZYP-5052 auto-induction medium at 37 °C with shaking at 200 rpm for 48 h. Thin-layer chromatography (TLC) was carried out on lipids extracted from 5 mg of dried cells. tDGAT, WS/DGAT from *T. curvata*, RoPAP, PAP from *R*. *opacus* PD630. RoFadD1, putative acyl-CoA synthetase from *R*. *opacus* PD630. FadR, fatty acid metabolism regulator from *E*. *coli* MG1655; RoTetR1/2/3, three putative fatty acid metabolism regulators from *R*. *opacus* PD630. All data are the means ± standard deviations from triplicates

To construct an acyl-CoA-dependent TAG pathway in *E. coli*, it is essential to enhance the acyl-CoA pool in addition to inducing RoPAP for DAG accumulation. Two putative acyl-CoA synthetases were predicted from oleaginous bacterium *R. opacus* PD630 and were designated as RoFadD1 (EHI43506) and RoFadD2 (AHK27355). Additional expression of *RoFadD1* along with *tDGAT* and *RoPAP* increased the cellular TAG titer, from 92.5 to 105.9 mg/L (Fig. 3). This suggested that there was a limited cytosolic acyl-CoA pool in engineered *E. coli*, and thus, activation of FFAs to their corresponding acyl-CoAs by the action of RoFaD1 enhanced TAG synthesis. FFAs were also boosted as the result of *RoFadD1* overexpression (Fig. 3). This may be explained by the other important function of acyl-CoA synthetase which is transportation of exogenous fatty acids across the inner membrane [23]. Alternatively, the increased TAGs and FFAs are due to the altered metabolic flux. The sub-location and detailed function of RoFadD1 await further investigations. In contrast, RoFadD2 did not show the ability to increase TAGs when compared with RoFadD1 (Additional file 4: Figure S4).

### Newly discovered fatty acid metabolism regulators from *R. opacus* PD630 contributed to the increased TAGs

RoTetR1/2/3 from the genome sequence of oleaginous *R. opacus* PD630 were denoted as three putative fatty acid metabolism regulators, although none of them contributed to the increased TFAs or hydroxyl fatty acids [32]. We tested whether they would enhance TAG synthesis. Results showed that expressing either *RoTetR* in addition to *tDGAT*, *RoPAP,* and *RoFadD1*, all led to increased TAG accumulation, with values of 149.7 mg/L (RoTetR1), 141.8 mg/L (RoTetR2), and 141.1 mg/L (RoTetR3), respectively (Fig. 3). The universal fatty acid metabolism regulator FadR slightly increased TAGs as well, rising from 105.9 mg/L (without regulator) to 119.5 mg/L (Fig. 3). FFAs were drastically reduced when either *RoTetR2* or *RoTetR3* was expressed alongside *tDGAT*, *RoPAP* and *RoFadD1* (Fig. 3), suggesting that most FFAs were converted to their respective acyl-CoAs under the tested conditions. Our previous result indicated that FadR increased the hydroxyl fatty acid production in *E. coli*, whereas none of the RoTetRs possessed the same ability [32]. Furthermore, only *fadR* overexpression stimulated the monounsaturated fatty acids C16:1 and C18:1 at the expense of saturated C14:0 in this study, similar to the results reported elsewhere (Additional file 5: Figure S5) [27]. Therefore, we predicted that the regulation mechanism of RoTetRs might be different from that of FadR. Further investigation of the differentially expressed genes regulated by each RoTetR would be helpful for identification of the regulation mechanism.

**Fig. 4:**
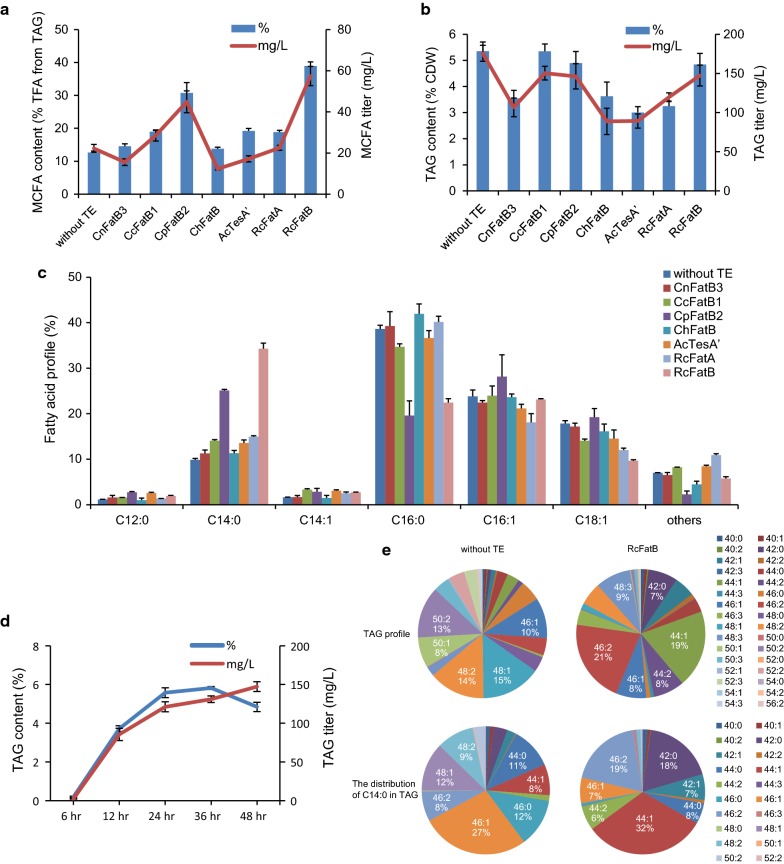
Assessment of medium-chain specific TE for diverting MCFA flux into TAG backbones. **a** MCFAs from the TAG fraction extracted from strain 2119 carrying different TEs. **b** TAG content and titer of strain 2119 carrying different TEs. **c** Fatty acid profile of the TAG fraction extracted from engineered strain 2119 carrying different TEs. **d** Time course of TAG production yielded by strain 2119 carrying pACYCDuet::*RcFatB*. Thin-layer chromatography (TLC) of lipid extracted from dried cells. **e** TAG profile and the distribution of C14 in the total TAGs from lipids extracted from strain 2119 and strain 2119 plus pACYCDuet::*RcFatB*, respectively. Strain 2119: *E*. *coli* BL21(DE3) carrying plasmid pCDFDuet::*tDGAT*/*RoPAP*::*RoFadD1*/*RoTetR2*. 48:1 represents 48 carbons with 1 double bond in three acyl chains. All data are the means ± standard deviations from triplicates

To alleviate the growth and metabolic burden, four selected genes including *tDGAT*, *RoPAP*, *RoFadD1,* and *RoTetR2* were next rearranged into the same pCDFDuet vector to form the plasmid pCDFDuet::*tDGAT*/*RoPAP*::*RoFadD1*/*RoTetR2* with the RBS motif upstream of each gene (Additional file 6: Figure S6). By expressing this plasmid in *E. coli* BL21(DE3) (designated as strain 2119), the TAG titer was further increased to 175.7 mg/L as expected (see results below, Fig. 4). We also observed that lowering the culturing temperature from 37 to 30 °C caused drastically decreased TAGs in strain 2119 (Additional file 7: Figure S7). Because tDGAT was isolated from a thermophilic actinobacterium with an optimal growing temperature of 50 °C [18]. Thus, it was likely that tDGAT exhibited higher activity at 37 °C compared to 30 °C.

### Overexpression of the castor TE gene *RcFatB*-triggered MCFA flux into TAG backbones

The next step was to selectively release MCFAs from the acyl-ACP pool and incorporate them into TAGs backbone. Six selected MCFA-specific TE genes including *CnFatB3*, *CcFatB1*, *CpFatB2*, *ChFatB2*, *AcTesA’,* and *RcFatB* were first inserted into the low copy number pACYCDuet-1 vector. Each TE was then expressed in strain 2119. Castor RcFatA was used as a control as it exhibited a preference for C16 and C18 instead of MCFAs. TLC combined with GC results showed that the RcFatB combination resulted in the highest MCFA titer (mainly C12:0, C14:0, and C14:1) in the form of TAG, which reached 57.4 mg/L. The increase in MCFA content from 12.7 to 39.0% in the TAGs was mainly at the expense of C16:0 (Fig. 4a, c). On the other hand, additional expression of *RcFatB* also caused a drop in the total amount of TAGs from 175.7 to 147.4 mg/L (Fig. 4b). Similar to the RcFatB combination, the CpFatB2 combination produced 27.9% MCFAs in the TAG, corresponding to 44.9 mg/L MCFAs in the form of TAG. Again, the total TAGs dropped to 146.2 mg/L in the CpFatB2 combination, similar to that of RcFatB (Fig. 4).

We also observed that the RcFatB combination yielded 140.2 mg/L MCFAs from cell lysate. However, only 41% of the MCFAs were incorporated into TAG backbones, suggesting that a large amount of MCFAs existed in the form of cellular FFA or membrane lipids. Accumulation of high levels of MCFA is known to be toxic to *E. coli* cells, since MCFAs would be partially incorporated into membrane lipids and would lead to impaired membrane functions [38]. We indeed observed that biomass in the RcFatB or CpFatB2 combinations was decreased with 17.9 and 25.8% reductions observed, respectively. Nevertheless, such a reduction may be an acceptable trade-off, since the value-added TAGs will be produced anyway, and the reductions may be minimized by optimization of nutritional levels. Besides, strategies to prevent the incorporation of MCFA into membrane lipid should be considered in the near future.

RcFatB has been reported as being active on oleoyl-ACP and palmitoyl-ACP in vitro [39]. However, the preference of RcFatB for myristoyl-ACP or lauroyl-ACP substrates was not tested in vitro. In another report, *RcFatB* was overexpressed in *E. coli* to produce 2.0 g/L FFAs at 48 h with 40% C14 [40]. The combined expression of *RcFatB* with other essential type II fatty acid synthesis genes has also been tested in *Saccharomyces cerevisiae*, leading to the production of 32% C14 fatty acids (20% C14:1 and 12% C14:0) among the TFAs [41]. Combining our results here, we predicted that RcFatB probably would exhibit a myristoyl-ACP preference in vitro, although there was only a trace amount of C14 accumulation in the seed of the RcFatB source plant, castor (*Ricinus communis* L).

The TAG production from combined expression of *RcFatB*, *tDGAT*, *RoPAP*, *RoFadD1*, and *RoTetR2* during the time course of the assay was then investigated. The TAG content increased to 5.8% (w/w CDW) after culturing for 36 h, while the highest TAG titer achieved was 147.4 mg/L at 48 h (Fig. 4d). The TAGs were accumulated in cells as early as 6 h, although the amount was quite low (Fig. 4d). This was consistent with the previous results, where TAGs were detected after induction of tDGAT expression for only 30 min [18]. Compared to oleaginous bacteria and yeasts, a shorter fermentation time was observed for TAG accumulation in engineered *E*. *coli*. This might be beneficial when seeking to produce TAGs in engineered *E. coli* cells.

Expression of *RcFatB* also significantly changed the TAG profile (Fig. 4e). With RcFatB, TAG-44:1 and TAG-46:2 accounted for 21 and 19% of the total TAG, respectively, suggesting that 16:1/14:0/14:0 or 16:0/14:1/14:0, and 16:1/16:1/14:0 might be the most abundant TAG classes. This result further confirmed the increased MCFA flux into TAG backbones. In addition, the distribution of MCFAs in TAG species was analyzed. The predominant C14:0 was mainly found in TAG-44:1, representing 32% of the total C14:0 containing TAGs, and indicating that 16:1/14:0/14:0 or 16:0/14:1/14:0 might be the most abundant TAGs for sequestration of C14:0 in the RcFatB combination. In contrast, C14:0 mainly existed in TAG-46:1 without RcFatB expression (Fig. 4f).

We also observed that the MCFAs dropped to control levels (12.5% of the total TAGs) if *RcFatB* was expressed by the high copy number plasmid pCDFDuet::*tDGAT*/*RoPAP*::*RoFadD1*/*RoTetR2/RcFatB,* although the total TAG and TFA titers further increased to 234.7 mg/L (7.0% CDW) and 553.2 mg/L (16.5% CDW), respectively (Additional file 6: Figure S6 and Additional file 8: S8). A similar result was observed in the RcFatA combination as well (Additional file 8: Figure S8). The increase in the total TAGs was probably caused by a lower metabolic burden or less MCFA toxicity. However, considering the drastically decreased MCFAs, pCDFDuet::*tDGAT*/*RoPAP*::*RoFadD1*/*RoTetR2/RcFatB* was not appropriate for MCFA production. Our previous results revealed that expression of *CnFatB3*, *CcFatB1,* or *CpFatB2* with low copy numbers rather than high copy number plasmids boosted the production of medium-chain hydroxyl fatty acids in *E. coli* [32]. It is assumed that the binary activities of TE towards both acyl-ACP and acyl-CoA resulted in diverting the acyl pools to FFA. The accumulation of MCFA-enriched TAGs might also depend on the efficiency of the interaction between generated acyl-ACPs and their corresponding applied TEs in engineered *E. coli*. Therefore, the choice of vector for expressing TEs was also crucial for the production of MCFAs. Further increases in TAG production may be achieved by balancing metabolic fluxes through expression of these genes under optimized promoters and/or RBS.

Collectively, castor RcFatB showed a preference for MCFAs mainly C14 fatty acids in *E. coli* cells, although C14:0 or C14:1 is not the major fatty acid in castor seed oils [42]. RcFatB may also be considered a good candidate for future production of tailored fatty acids and their derivatives. Furthermore, the results also demonstrated that tDGAT was able to transfer acyl-CoA with tailored fatty acids into TAG backbones, although the efficiency was undetermined.

### TAG induction through nutrient-level optimization

**Fig. 5:**
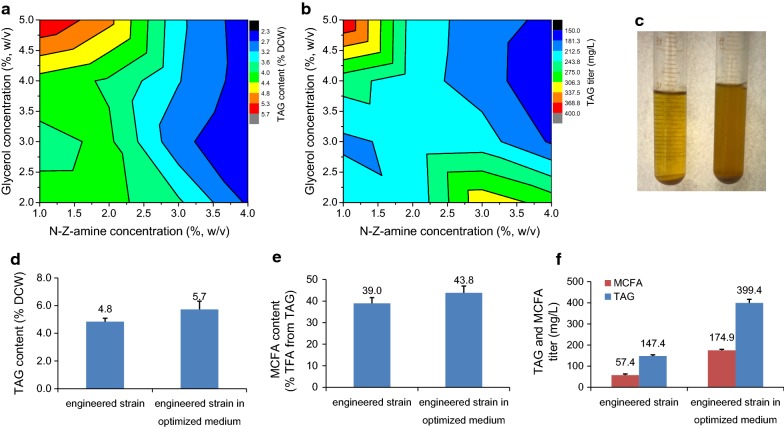
TAG induction through nutrient level optimization. Contour profiles of TAG content (**a**) and TAG titer (**b**) for strain 2119 harboring pACYCDuet::*RcFatB* cultured in 16 designed media with different ratios of glycerol and N-Z-amine. Floating cells were observed during the collection of cells (**c**). Increases in the TAG/MCFA content (**d**, **e**) and titer (**f**) realized from nutrient optimization. The detailed information on designed media is shown in Additional file 10: Table S1. Cells were cultured at 37 °C with shaking at 200 rpm for 48 h. All data are the means ± standard deviations from triplicates

We next sought to increase the TAG titer without decreasing the MCFA proportions by optimization of nutrient levels. Auto-induction medium ZYP-5052 was shown to be more suitable for TAG accumulation in *E. coli* than conventional LB medium with IPTG induction [16]. However, ZYP-5052 was originally designed for high expression of any target protein under the T7 promoter. Thus, it was probably not the best choice for TAG accumulation. Therefore, the ZYP-5052 medium was optimized in this study for improving TAG synthesis (Additional file 10: Table S1). The results here suggested that the ratio of glycerol to N-Z-amine, rather than the absolute concentration of glycerol or N-Z-amine, was crucial towards enhancing both the TAG and MCFA titers (Fig. 5a, b). After optimization, the highest TAG titer was achieved of up to 5.7% (w/w CDW) with 43.8% of MCFAs, corresponding to 399.4 mg/L of total TAGs (Fig. 5d, e). The optimized medium containing 1% N-Z-amine, 5% glycerol, 25 mM (NH_4_)_2_SO_4_, 3 mM MgSO_4_, 0.5% aspartic acid, and 0.5% serine, and the other ingredients were the same as in ZYP-5052. The TAG titer and content were both elevated as the supplementation with glycerol increased (Fig. 5). Glycerol as a renewable by-product can be channeled into glycolysis to provide a substrate for fatty acids, membrane lipids, and DAG synthesis. More importantly, unlike glucose, the increased glycerol concentration would not prevent induction by lactose in auto-induction media [43]. As to the effect of nitrogen N-Z-amine, despite the fact that increasing nitrogen levels improved the biomass, the TAG content was drastically decreased (Fig. 5a). In addition, neither a change in the NH_4_^+^/Mg^2+^ dosage nor supplementation with aspartic acid and serine caused an obvious TAG change.

Large amounts of floating cells were also observed during the collection process, especially for cells cultured in the optimized medium. This indicated that these cells had high buoyancy and thus could float in the medium once the culture was settled for 3 h, whereas normal cells settled to the bottom of the tube (Fig. 5c). A similar observation has been described in the oleaginous yeast, *Yarrowia lipolytica*, which also produces high levels of lipids [44]. The floating characteristics of such engineered cells may facilitate the subsequent purification process.

### Small and membrane-bound lipid droplets (LD) were formed in recombinant *E. coli*

**Fig. 6:**
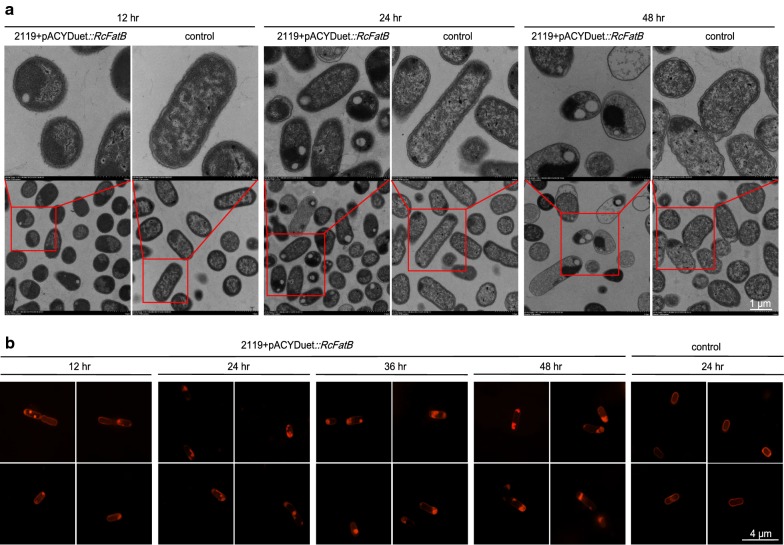
Observation of LDs in engineered *E. coli* cells. Cells were observed by transmission electron microscopy (TEM) (**a**) and N-SIM super-resolution microscope (**b**). The engineered strain 2119 carrying pACYCDuet::*RcFatB* cultured in ZYP-5052 auto-induction medium at 37 °C with shaking at 200 rpm for 12, 24, 36 or 48 h. LDs were stained with Nile Red before observing by N-SIM. The scale bar is 1 µm for TEM and 4 µm for N-SIM

We next investigated whether TAGs rich in MCFAs could be accumulated in the form of LDs. Both N-SIM super-resolution microscopy and TEM analysis clearly showed that small LDs were formed and accumulated in cells after culturing for only 12 h in optimized medium (Fig. 6). After culturing for 24 h or more, large LDs with irregular shapes were observed in cells, suggesting that LDs were fused via an unknown mechanism in late stationary cells (Fig. 6). We also noticed that almost all observed LDs were attached to cell membranes (Fig. 6). Prokaryotic LDs have been considered to be phase-separated organelles in the cytoplasm, and their formation starts with attachment of WS/DGAT to the cytoplasm membrane [45]. Unlike eukaryotic LDs, prokaryotic LDs have been predicted as being surrounded by a monolayer of phospholipids [45, 46]. The observed membrane-bound LDs from engineered *E. coli* cells were consistent with earlier predictions. In yeast cells, if storage lipids such as TAGs are not generated from inert end product pools, they would be metabolically active [47]. However, this was not the case in the engineered *E. coli*, as most of the observed LDs were still intact in the lysed cells after culturing for 48 h (Fig. 6b). This characteristic might be beneficial for further purification of prokaryotic LDs.

Despite the fact that many important proteins contribute to LD formation or stability, for example oleosin, lipid droplet associated protein (LDAP) and caleosin, all of which have been identified and characterized from oil plants, few functional proteins have been revealed from oleaginous bacteria [48]. However, one such representative protein is TadA from *R. opacus* PD630, and mutation of TadA has led to 30–40% less TAGs and smaller LDs when compared to the parental strain [49]. However, we did not find any homologues of TadA in *E. coli* and overexpression of *TadA* dramatically decreased the TAG titer in the engineered TAG-producing strain 2119 (Additional file 9: Figure S9). Furthermore, the Ro02104 protein isolated from *R. jostii* RHA1 also plays a role in LD dynamics [50]. Recently, an LD-associated protein MLDS from *R. jostii* RHA1 was demonstrated to mediate binding of LDs to genomic DNA and thus enhance the cell survival under stress [51]. Whether the LDs generated by engineered *E. coli* could exert similar roles is unknown. Nevertheless, the formation of LDs did not cause obvious cell lysis or impair cell growth in our study. It is presumed that identification of the mechanism involved in prokaryotic LD formation and characterization of LD-associated proteins may facilitate the stability of LDs or further enhance the TAG titer in engineered strains. Collectively, TAGs with tailored fatty acids can be stored in the LDs of engineered *E. coli*. Such prokaryotic LDs may be exploited for various biotechnology applications due to their simple yet robust structures, as well as their amenability to extraction.

## Conclusions

In conclusion, we reconstructed an acyl-CoA-dependent pathway in *E. coli* by introducing four heterologous genes including *tDGAT*, *RoPAP*, *RoFadD1,* and *RoTetR2* to produce high levels of TAG. Co-expression of castor *RcFatB* with the above genes created MCFA substrates available for TAG assembly. The titers of TAGs rich in MCFAs reached 399.4 mg/L, attaining their highest levels under shake flask conditions. In addition, membrane-bound LDs were formed in engineered *E. coli*. This illustrated that *E. coli* as a prokaryotic cell factory has the potential to produce value-added TAG.

## Methods

### Chemicals, enzymes, and kits

Chemicals including primuline, hexane, acetone, acetic acid, and diethyl ester were purchased from Sigma-Aldrich (St. Louis, USA) or Sinopharm (Shanghai, China). Methanol, chloroform, Phusion polymerase, restriction enzymes, T4 DNA ligase, DNase I, and RevertAid First Strand cDNA Synthesis Kit were purchased from Thermo Fisher Scientific (Beverly, USA). Plasmid Miniprep Kit, Total DNA Extraction Kit, PCR Purification Kit, and Gel Extraction Kit were purchased from TianGen (Beijing, China). RNA Protect Reagent and RNeasy Mini Kit were purchased from Qiagen (Hilden, Germany). *Power* SYBR^®^ Green PCR Master Mix was from Applied Biosystems (Massachusetts, USA). FFA, DAG, TAG, or FAEE standards were purchased from Nu-Chek (Elysian, USA) or Larodan (Stockholm, Sweden).

### Strains, media, and growth conditions

*Escherichia coli* DH5α was used for plasmid construction and propagation. *E. coli* BL21(DE3) was used for gene expressing and TAG production. Cells were cultivated in Luria–Bertani medium (LB, 1% (w/v) tryptone, 0.5% (w/v) yeast extract, and 1% (w/v) NaCl) at 37 °C with shaking at 200 rpm for cell inoculation or plasmid propagation. Appropriate antibiotics were added at the following concentrations: kanamycin 50 mg/L, ampicillin 100 mg/L, spectinomycin 50 mg/L, or chloramphenicol 34 mg/L, respectively. Auto-induction medium ZYP-5052 with appropriate antibiotics was mainly used for TAG production [43]. For time-course experiment, cells were collected after culturing in ZYP-5052 at 37 °C with shaking at 200 rpm for 6, 12, 24, 36, and 48 h, respectively.

For nutrient optimization, 16 designed media were tested for TAG production. The detailed information of the designed media is listed in Additional file 10: Table S1. Cells were cultured at 37 °C with shaking at 200 rpm for 48 h.

### Plasmid construction and gene expression

All constructed strains and plasmids used in this study are summarized in Additional file 11: Table S2. Oligonucleotides used as PCR primers are listed in Additional file 13: Table S3. PAP encoding genes *RoPAP* (WP_005246202) from *R. opacus* PD630 and *RjPAP* (WP_011593404) from *R. Jostii* RHA1 were chemically synthesized and inserted into pCDFDuet-1, respectively. Then, each gene was subcloned into the downstream of *atfA* (AAO17391, from *A. baylyi* ADP1) flanked by a Ribosome-Binding Site (RBS) sequence (AAGGAG). Other WS/DGAT encoding genes include *atf1* (EHI42943) and *atf2* (EHI41112) from *Rhodococcus opacus* PD630, *atf8* (ACY38595) from *R. jostii* RHA1, *tDGAT* (WP_012854133) from *Thermomonospora curvata* DSM 43183, and *atfA_co* (*atfA* was codon optimized for *E. coli* expression). Each above WS/DGAT encoding gene was chemically synthesized and subcloned into upstream of *RoPAP* flanked by a RBS sequence, located in the *Eco*RI/*Not*I sites of pCDFDuet-1. Acyl-CoA synthetase genes *RoFadD1* (EHI43506) or *RoFadD2* (AHK27355), from *R. opacus* PD630, were then inserted into the *Nde*I*/Xho*I located at the second multiple cloning sites (MCS2) of pCDFDuet-1. Fatty acid metabolism regulators *fadR* from *E. coli*, or *RoTetR1*, *RoTetR2*, and *RoTetR3* from *R*. *opaque* PD630 were subcloned into pET28a in our previous work [32]. For assembling multiple genes into one vector, *tDGAT* and *RoPAP*, flanked by RBS were introduced into *Eco*RI/*Not*I sites of pCDFDuet-1, while *RoFadD1* and *RoTetR2* flanked by RBS were inserted into *Nde*I*/Xho*I sites, generating the plasmid pCDFDuet::*tDGAT*/*RoPAP*::*RoFadD1*/*RoTetR2*. The detailed information on constructed plasmids is shown in Additional file 6: Figure S6.

TE genes *RcFatA* (NP_001310680) and *RcFatB* (NP_001310677) from *Ricinus communis*, *ChFatB2* (AAC49269) from *C*. *hookeriana*, *AcTesA’* from *A. baylyi* were all codon-optimized, chemically synthesized, and inserted into pACYCDuet-1, respectively. Three other plant TE genes, *CnFatB3*, *CcFatB1,* and *CpFatB2*, previously constructed in pACYCDuet-1 vector [32], were also used in this study. The gene *RcFatA* or *RcFatB* with RBS at 5′-end was also inserted into pCDFDuet::*tDGAT*/*RoPAP*::*RoFadD1*/*RoTetR2* via seamless assembly technology to generate pCDFDuet::*tDGAT*/*RoPAP*::*RoFadD1*/*RoTetR2/RcFatA or* pCDFDuet::*tDGAT*/*RoPAP*::*RoFadD1*/*RoTetR2/RcFatB*, respectively (Additional file 6: Figure S6).

### Lipid analysis

*Escherichia coli* cells were cultured each in 50 mL medium, and the mass of the freeze-dried cells was measured. Total lipids were isolated from two and half or five milligram of the freeze-dried *E. coli* cells according to a previous method [16]. The extracted lipids were separated by thin-layer chromatography (TLC) using a solvent system (hexane:diethyl ester:acetic acid = 70:30:1), and visualized by spraying with 0.01% primuline dissolved in 80% acetone. The bands corresponding to FFA and TAG were recovered from TLC plates and methylated as previously described [1]. The resulting fatty acid methyl esters (FAMEs) were then analyzed using a 7890A gas chromatography (GC, Agilent technologies, USA) equipped with a flame ionization detector (FID) and an HP-FFAP column (30 m × 250 µm i.d. × 0.25 µm thickness). For quantification, either 10 µg of C15:0-TAG and 5 µg of C15:0-FFA (for low levels of TAG or FFA) or 20 µg of C15:0-TAG and 10 µg of C15:0-FFA were added into samples as internal standards before lipid extraction, respectively. Lipids from 2 mg dried cells were directly methylated to FAMEs and analyzed by GC as described above, to quantify cellular TFA.

Shotgun lipidomic analysis of TAG profile was done by liquid chromatography–mass spectrometry (LC–MS) [52]. Briefly, a hybrid triple quadrupole/linear IT mass spectrometer, API 4000 Q-Trap (AB SCIEX, Foster City, CA, USA), operating under an Analyst software system was utilized here. The heater temperature along the ion transfer capillary was maintained at 300 °C. The shelter gas (nitrogen) pressure was 344.75 kPa. The diluted lipid (0.01–0.06 mg/mL) was directly infused into the ESI source at a flow rate of 0.02 mL/min. TAG profile was analyzed with tools at the website: http://www.lipidmaps.org.

### Microscopy analysis

For transmission electron microscopy (TEM) imaging, cells were fixed with 2.5% glutaraldehyde and 2% paraformaldehyde in 0.1 M phosphate buffer pH7.4 under vacuum for 24 h. For super-resolution microscopy observation, aliquots (200 µL) of cell cultures grown for 12, 24, 36, or 48 h were harvested, washed and dissolved in phosphate-buffered saline (PBS), and then stained with 2 µL of Nile Red (1 mg/mL in acetone stock) for 30 min in the dark. Stained cells were washed three times with PBS and then suspended in 100 µL PBS. Samples were observed immediately using a Structured Illumination Microscope (N-SIM, Nikon, Japan) equipped with an EMCCD camera (iXon DU-897, Andor, Northern Ireland) and a 100 × 1.49 NA TIRF objective (CFI Apo TIRF, Nikon, Japan). The fluorescence was excited with a 561 nm laser and detected by a 570–640 nm bandpass emission filter. Images were captured in multiple phases and angles of the illumination pattern and then directly reconstructed with the Nikon NIS-Elements Ar software. The final pixel size of SIM image was 30 nm.

## Additional files

**Additional file 1: Figure S1.** Sequence alignment of five bacterial WS/DGATs. tDGAT: WS/DGAT from *T. curvata*; AtfA: WS/DGAT from *A. baylyi* ADP1; Atf8: WS/DGAT from *R. jostii* RHA1; Atf1/Atf2: WS/DGATs from *R. opacus* PD630. The predicted catalytic motif “HHxxxDG”, motif I “PLW” and motif II “ND” were indicated by arrows.

**Additional file 2: Figure S2.** Comparison of three bacterial phosphatidic acid phosphatases (PAPs). A, sequence alignment of bacterial PAPs. B, TFAs produced by the engineered cells harboring empty vector pCDFDuet-1, *RjPAP* or *RoPAP*, respectively. C, fatty acid profile of the cellular fatty acids from the engineered *E. coli*. PgpB, PAP from *E. coli* MG1655; RoPAP, PAP from *R. opacus* PD630; RjPAP, PAP from *R. jostii* RHA1. All data are the means ± standard deviations from triplicates.

**Additional file 3: Figure S3.** Comparison of primuline (A) and iodine (B) staining of neutral lipids extracted from the engineered *E. coli* cells.

**Additional file 4: Figure S4.** A putative acyl-CoA synthetase RoFadD2 from *R. opacus* PD630 did not increase TAG. Cells were cultured in ZYP-5052 auto-induction medium at 37 °C with shaking at 200 rpm for 48 hr. Lipids extracted from 2.5 mg dried cells were loaded on TLC plate.

**Additional file 5: Figure S5.** Fatty acid profile of extracted TAGs from engineered *E. coli* BL21(DE3) strains harboring different gene combinations. tDGAT: WS/DGAT from *T. curvata*; RoPAP: PAP from *R. opacus* PD630; RoFadD1: putative acyl-CoA synthetase from *R. opacus* PD630; RoTetR1/2/3: three putative fatty acid metabolism regulators from *R. opacus* PD630; FadR: fatty acid metabolism regulator from *E. coli* MG1655. All data are the means ± standard deviations from triplicates.

**Additional file 6: Figure S6.** The detailed information on representative constructed plasmids.

**Additional file 7: Figure S7.** Effect of the incubation temperature on TAG synthesis. Strain 2119 was cultured in ZYP-5052 auto-induction mediumat 30 or 37 °C with shaking at 200 rpm for 48 hr. Strain 2119, *E.coli* BL21(DE3) harboring pCDFDuet::*tDGAT*/*RoPAP*::*RoFadD1*/*RoTetR2*.

**Additional file 8: Figure S8.** One-vector or two-vector system used for the expression of castor acyl-ACP thioesterase genes *RcFatA* and *RcFatB*. The total TAG content and titer (A), or the TFA content and titer (B) from four engineered *E.coli* strains. 2119+pACY-RcFatA, strain 2119 harboring pACYCDuet::*RcFatA*; 2119+pACY-RcFatB, strain 2119 harboring pACYCDuet::*RcFatB*. 2119-RcFatA, *E. coli* BL21(DE3) harboring pCDFDuet::*tDGAT*/*RoPAP*::*RoFadD1*/*RoTetR2/RcFatA.* 2119-RcFatB, *E.coli* BL21(DE3) harboring pCDFDuet::*tDGAT*/*RoPAP*::*RoFadD1*/*RoTetR2/RcFatB.* Strain 2119, *E.coli* BL21(DE3) harboring pCDFDuet::*tDGAT*/*RoPAP*::*RoFadD1*/*RoTetR2*. All data are the means ± standard deviations from triplicates.

**Additional file 9: Figure S9.** Overexpression of an LD-associated protein-encoding gene *TadA* decreased the TAG titer in strain 2119. Cells were cultured in ZYP-5052 auto-induction medium at 37°C with shaking at 200 rpm for 48 hr. Lipids extracted from 5 mg dried cells were loaded on TLC plate. Strain 2119, *E.coli* BL21(DE3) harboring pCDFDuet::*tDGAT*/*RoPAP*::*RoFadD1*/*RoTetR2*.

**Additional file 10: Table S1.** 16 media were designed based on the ZYP-5052 auto-induction medium for improving the accumulation of TAGs rich in MCFAs.

**Additional file 11: Table S2.** Strains and plasmids used in this study.

**Additional file 12: Table S3.** Primers used in this study.

## Abbreviations

MCFA: medium-chain fatty acids
CDW: cell dry weight
DAG: diacylglycerol
WS/DGAT: wax ester synthase/acyl-Coenzyme A:diacylglycerol acyltransferase
FAME: fatty acid methyl ester
FFA: free fatty acid
TFA: total fatty acid
SCO: single-cell oil
TAG: triacylglycerol
acyl-ACP: acyl-acyl carrier protein
TE: acyl-ACP thioesterase
TLC: thin-layer chromatography
FAEE: fatty acid ethyl ester
LD: lipid droplet
