# Supplementary material for: Stepwise metabolic engineering of Escherichia coli to produce triacylglycerol rich in medium-chain fatty acids
Source: Biotechnol Biofuels. 2018 Jun 25;11:177. doi: 10.1186/s13068-018-1177-x (PMC6016142; doi:10.1186/s13068-018-1177-x)
Supplement: Supplementary file 1 — Additional file 1: Figure S1. Sequence alignment of five bacterial WS/DGATs. tDGAT: WS/DGAT from T. curvata; AtfA: WS/DGAT from A. baylyi ADP1; Atf8: WS/DGAT from R. jostii RHA1; Atf1/Atf2: WS/DGATs from R. opacus PD630. The predicted catalytic motif “HHxxxDG”, motif I “PLW” and motif II “ND” were indicated by arrows. [file 13068_2018_1177_MOESM1_ESM.pdf]

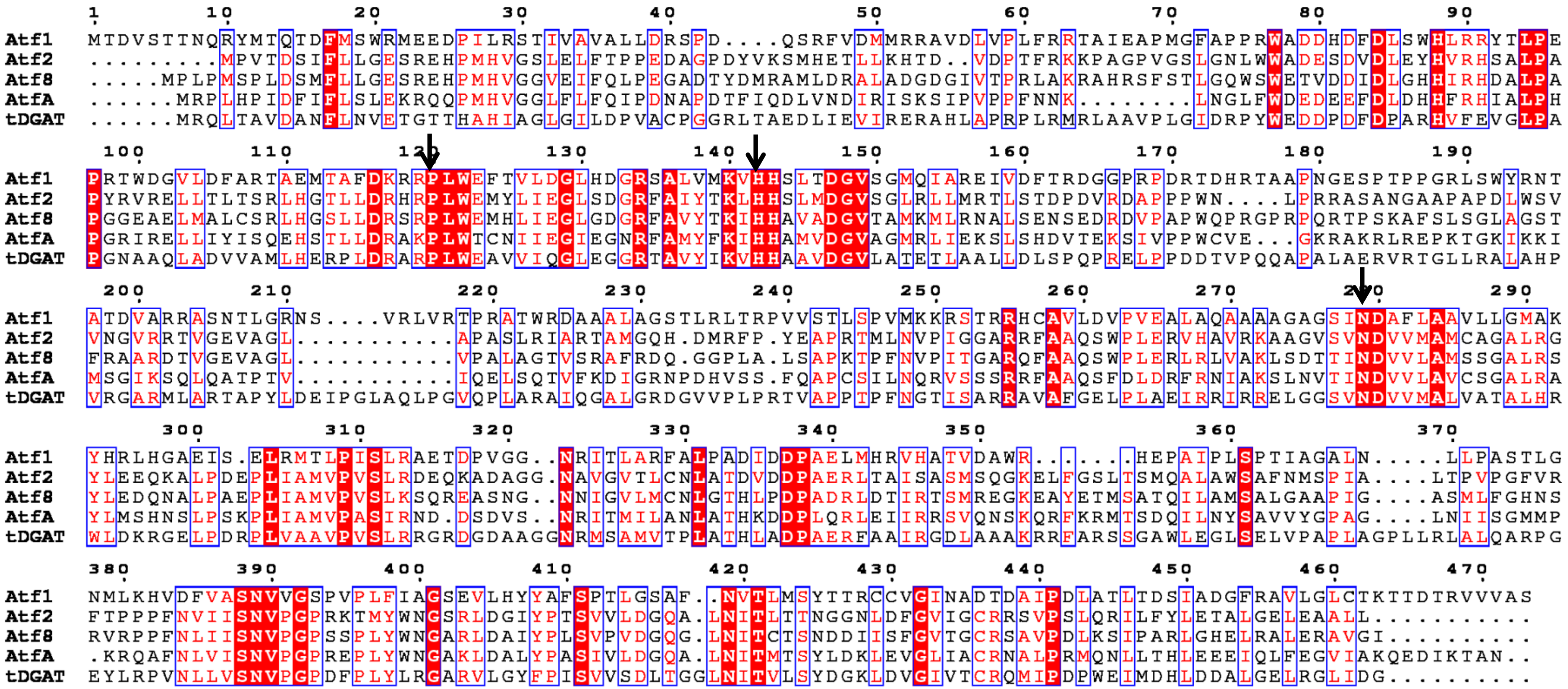
1 10 20 30 40 50 60 70 80 90
Atf1 MTDVSTTNQRYMTQTDFMSWRMEEDPILRSTIVAVALLDRSPD....QSRFVDMMRRAVDLVPLFRRTAIEAPMGFAPPRWADDHDFDLSWHLRRYTLPE
Atf2 .........MPVTDSIFLLGESREHPMHVGSLELFTPPEDAGPDYVKSMHETLLKHTD..VDPTFRKKPAGPVGSLGNLWWADESDVDLEYHVRHSALPA
Atf8 .....MPLPMSPLDSMFLLGESREHPMHVGGVEIFQLPEGADTYDMRAMLDRALADGDGIVTPRLAKRAHRSFSTLGQWSWETVDDIDLGHHIRHDALPA
AtfA ......MRPLHPIDFIFLSLEKRQQPMHVGGLFLFQIPDNAPDTFIQDLVNDIRISKSIPVPPFNNK.........LNGLFWDEDEEFDLDHHFRHIALPH
tDGAT ......MRQLTAVDANFLNVETGTTHAHIAGLGILDPVACPGGRLTAEDLIEVIRERAHLAPRPLRMRLAAVPLGIDRPYWEDDPDFDPARHVFEVGLPA
100 110 120 130 140 150 160 170 180 190
Atf1 PRTWDGVLDFARTAEMTAFDKRRPLWEFTVLDGLHDGRSALVMKVHHSLTDGVSGMQIAREIVDFTRDGGPRPDRTDHRTAAPNGESPTPPGRLSWYRNT
Atf2 PYRVRELLTLTSRLHGTLLDRHRPLWEMYLIEGLSDGRFAIYTKLHHSLMDGVSGLRLLMRTLSTDPDVRDAPPPWN....LPRRASANGAAPAPDLWSV
Atf8 PGGEAELMALCSRLHGSLLDRSRPLWEMHLIEGLGDGRFAVYTKIHHAVADGVTAMKMLRNALSENSEDRDVPAPWQPRGPRPQRTPSKAFSLSGLAGST
AtfA PGRIRELLIYISQEHSTLLDRAKPLWTCNIIEGIEGNRFAMYFKIHHAMVDGVAGMRLIEKSLSHDVTEKSIVPPWCVE...GKRAKRLREPKTGKIKKI
tDGAT PGNAAQLADVVAMLHERPLDRARPLWEAVVIQGLEGGRTAVYIKVHHAAVDGVLATETLAALLDLSPQPRELPPDDTVPQQAPALAERVRTGLLRALAHP
200 210 220 230 240 250 260 270 280 290
Atf1 ATDVARRASNTLGRNS....VRLVRTPRATWRDAAALAGSTLRLTRPVVSTLSPVMKKRSTRRHCAVLDVPVEALAQAAAGAGSINDAFLAAVLLGMAK
Atf2 VNGVRRTVGEVAGL...........APASLRIARTAMGQH.DMRFP.YEAPRTMLNVPIGGARRFAAQSWPLERVHAVRKAAGVSVNDVVMAMCAGALRG
Atf8 FRAARDTVGEVAGL...........VPALAGTVSRAFRDQ.GGPLA.LSAPKTPFNVPITGARQFAAQSWPLERLRLVAKLSDTTINDVVLAMSSGALRS
AtfA MSGIKSQLQATPTV...........IQELSQTVFKDIGRNPDHVSS.FQAPCSILNQRVSSSRRFAAQSFDLDRFRNIAKSLNVTINDVVLAVCSGALRA
tDGAT VRGARMLARTAPYLDEIPGLAQLPGVQPLARAIQGALGRDGVVPLPRTVAPPTPFNGTISARRAVAFGELPLAEIRRIRRELGGSVNDVVMALVATALHR
300 310 320 330 340 350 360 370
Atf1 YHRLHGAEIS.ELRMTLPISLRAETDPVGG..NRITLARFALPADIDDPAELMHRVHATVDAWR.......HEPAIPLSPTIAGALN.....LLPASTLG
Atf2 YLEEQKALPDEPLIAMVPVSLRDEQKADAGG.NAVGVTLCNLATDVDDPAERLTAISASMSQGKELFGSLTSMQALAWSAFNMSPIA....LTPVPGFVR
Atf8 YLEDQNALPAEPLIAMVPVSLKSQREASNG..NNIGVLMCNLGTHLPDPADRLDTIRTSMREGKEAYETMSATQILAMSALGAAPIG....ASMLFGHNS
AtfA YLMSHNSLPSKPLIAMVPASIRND.DSDVS..NRITMILANLATHKDDPLQRLEIIRRSVQNSKQRFKRMTSDQILNYSAVVYGPAG....LNIISGMMP
tDGAT WLDKRGELPDRPLVAAVPVSLRRGRDGDAAGGNRMSAMVTPLATHLADPAERFAAIRGDLAAAKRRFARSSGAWLEGLSELVPAPLAGPLLRLALQARPG
380 390 400 410 420 430 440 450 460 470
Atf1 NMLKHVDFVASNVVGSPVPLFIAGSEVLHYYAFSPTLGSAF..NVTLMSYTTRCCVGINADTDAIPDLATLTDSIADGFRAVLGLCTKTTDTRVVVAS
Atf2 FTPPPFNVIISNVPGPRKTMYWNGSRLDGIYPTSVVLDGQA.LNITLTTNGGNLDFGVIGCRRSVPSLQRILFYLETALGELEAAL..........
Atf8 RVRPPFNLIISNVPGPSSPLYWNGARLDAIYPLSVPVDGQG.LNITCTSNDDIISFGVTGCRSAVPDLKSIPARLGHELRALERAVGI.........
AtfA .KRQAFNLVISNVPGPREPLYWNGAKLDALYPASIVLDGQA.LNITMTSYLDKLEVGLIACRNALPRMQNLLTHLEEEIQLFEGVIAKQEDIKTAN..
tDGAT EYLRPVNLLVSNVPGPDFPLYLRGARVLGYFPISVVSDLTGGLNITVLSYDGKLDVGIVTCRQMIPDPWEIMDHLDDALGELRGLIDG.........
