# Supplementary figures and images for: Stepwise metabolic engineering of Escherichia coli to produce triacylglycerol rich in medium-chain fatty acids
Source: Biotechnol Biofuels. 2018 Jun 25;11:177. doi: 10.1186/s13068-018-1177-x (PMC6016142; doi:10.1186/s13068-018-1177-x)

A

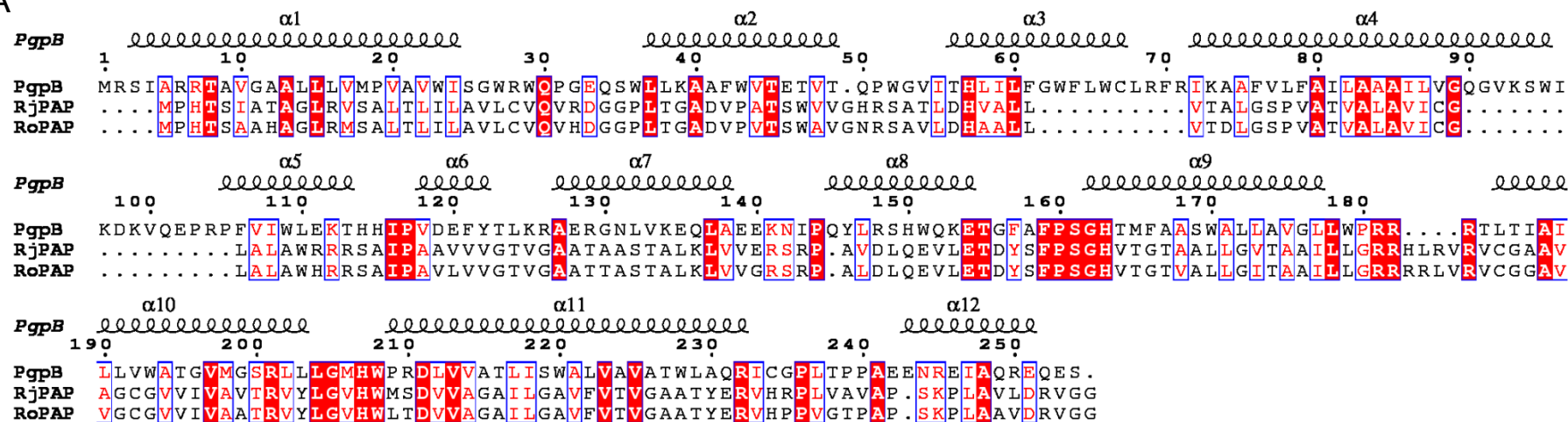

B

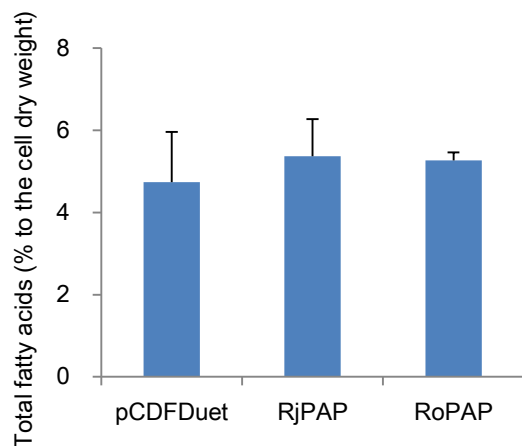

C

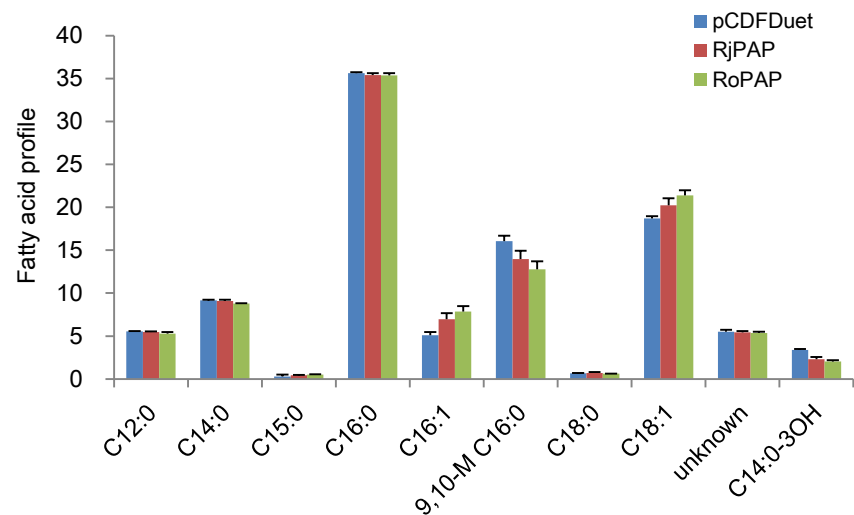

Supplement: Supplementary file 2 — Additional file 2: Figure S2. Comparison of three bacterial phosphatidic acid phosphatases (PAPs). A, sequence alignment of bacterial PAPs. B, TFAs produced by the engineered cells harboring empty vector pCDFDuet-1, RjPAP or RoPAP, respectively. C, fatty acid profile of the cellular fatty acids from the engineered E. coli. PgpB, PAP from E. coli MG1655; RoPAP, PAP from R. opacus PD630; RjPAP, PAP from R. jostii RHA1. All data are the means ± standard deviations from triplicates. [file 13068_2018_1177_MOESM2_ESM.pdf]

A

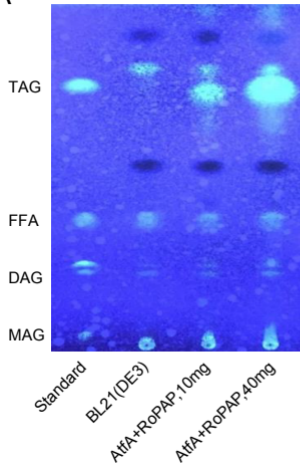

B

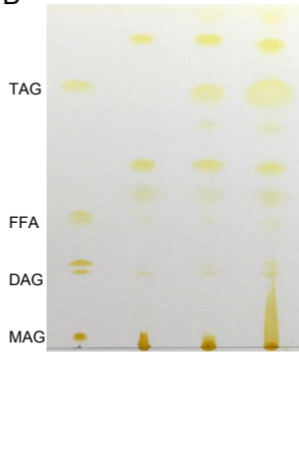

Supplement: Supplementary file 3 — Additional file 3: Figure S3. Comparison of primuline (A) and iodine (B) staining of neutral lipids extracted from the engineered E. coli cells. [file 13068_2018_1177_MOESM3_ESM.pdf]

TAG

standard  
tDGAT+RoPAP+RofadD1  
tDGAT+RoPAP+RofadD2  
tDGAT+RoPAP

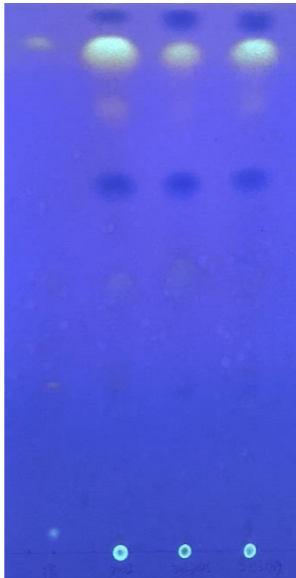

Supplement: Supplementary file 4 — Additional file 4: Figure S4. A putative acyl-CoA synthetase RoFadD2 from R. opacus PD630 did not increase TAG. Cells were cultured in ZYP-5052 auto-induction medium at 37 °C with shaking at 200 rpm for 48 hr. Lipids extracted from 2.5 mg dried cells were loaded on TLC plate. [file 13068_2018_1177_MOESM4_ESM.pdf]

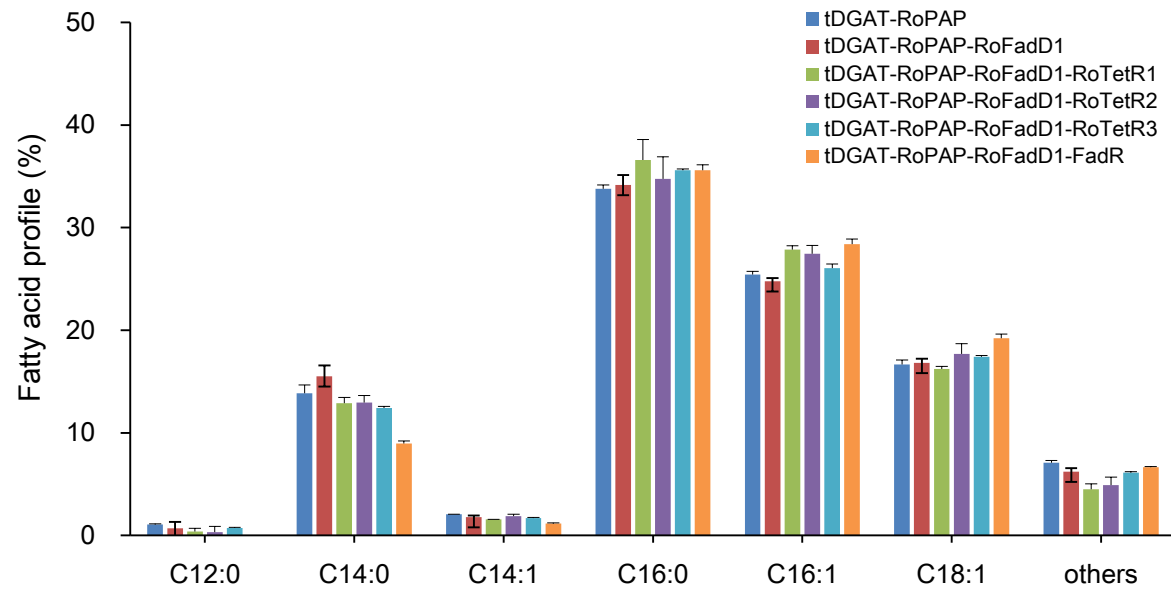

Supplement: Supplementary file 5 — Additional file 5: Figure S5. Fatty acid profile of extracted TAGs from engineered E. coli BL21(DE3) strains harboring different gene combinations. tDGAT: WS/DGAT from T. curvata; RoPAP: PAP from R. opacus PD630; RoFadD1: putative acyl-CoA synthetase from R. opacus PD630; RoTetR1/2/3: three putative fatty acid metabolism regulators from R. opacus PD630; FadR: fatty acid metabolism regulator from E. coli MG1655. All data are the means ± standard deviations from triplicates. [file 13068_2018_1177_MOESM5_ESM.pdf]

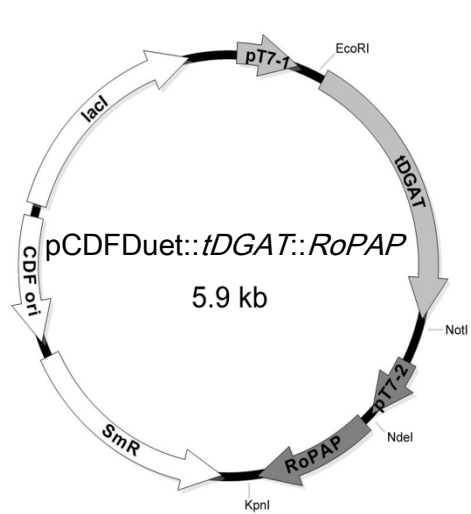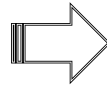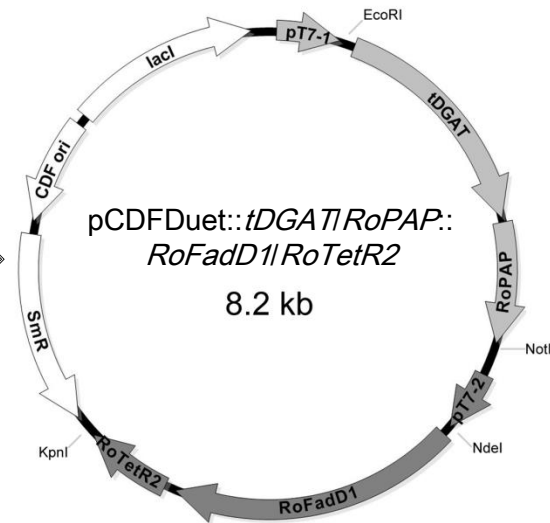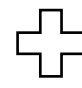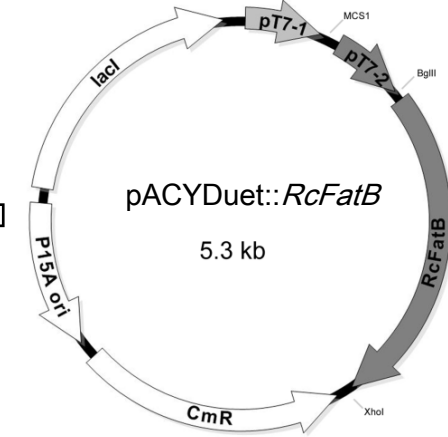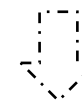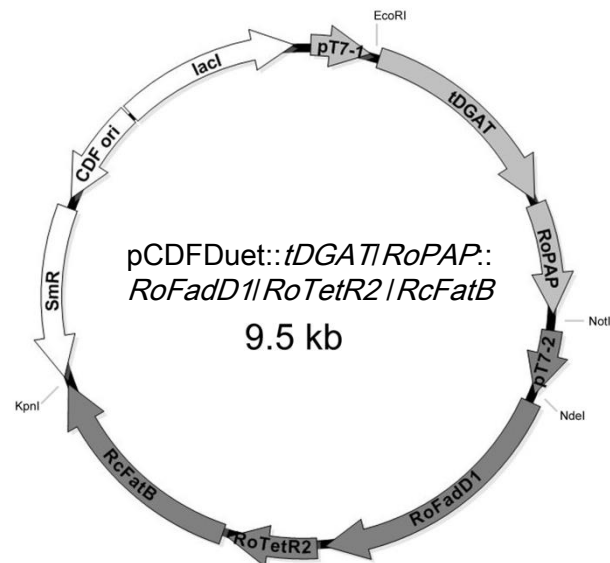

Supplement: Supplementary file 6 — Additional file 6: Figure S6. The detailed information on representative constructed plasmids. [file 13068_2018_1177_MOESM6_ESM.pdf]

TAG

FFA

DAG

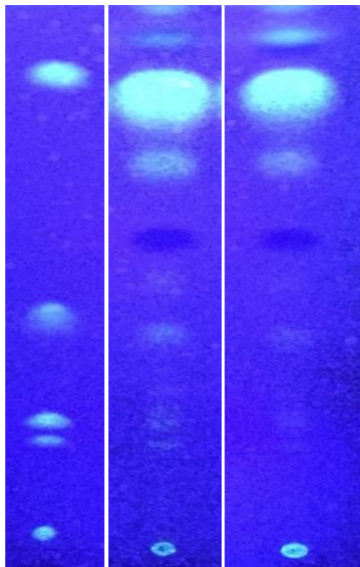

37°C

30°C

Supplement: Supplementary file 7 — Additional file 7: Figure S7. Effect of the incubation temperature on TAG synthesis. Strain 2119 was cultured in ZYP-5052 auto-induction mediumat 30 or 37 °C with shaking at 200 rpm for 48 hr. Strain 2119, E.coli BL21(DE3) harboring pCDFDuet::tDGAT/RoPAP::RoFadD1/RoTetR2. [file 13068_2018_1177_MOESM7_ESM.pdf]

A

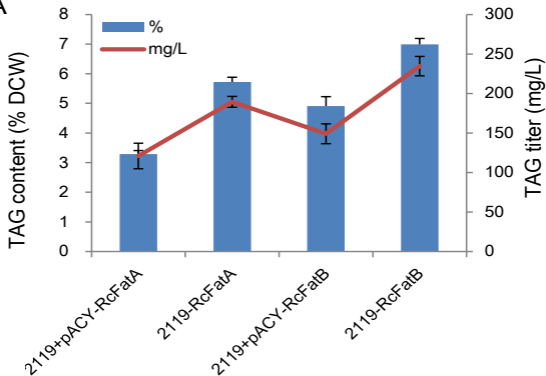

B

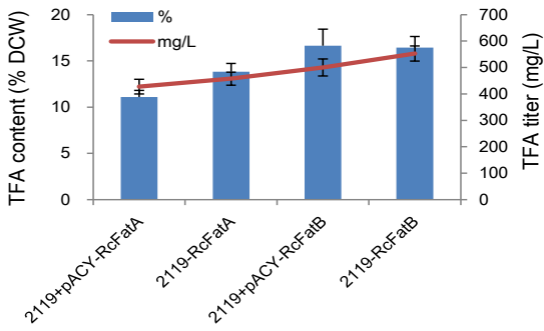

Supplement: Supplementary file 8 — Additional file 8: Figure S8. One-vector or two-vector system used for the expression of castor acyl-ACP thioesterase genes RcFatA and RcFatB. The total TAG content and titer (A), or the TFA content and titer (B) from four engineered E.coli strains. 2119+pACY-RcFatA, strain 2119 harboring pACYCDuet::RcFatA; 2119+pACY-RcFatB, strain 2119 harboring pACYCDuet::RcFatB. 2119-RcFatA, E. coli BL21(DE3) harboring pCDFDuet::tDGAT/RoPAP::RoFadD1/RoTetR2/RcFatA. 2119-RcFatB, E.coli BL21(DE3) harboring pCDFDuet::tDGAT/RoPAP::RoFadD1/RoTetR2/RcFatB. Strain 2119, E.coli BL21(DE3) harboring pCDFDuet::tDGAT/RoPAP::RoFadD1/RoTetR2. All data are the means ± standard deviations from triplicates. [file 13068_2018_1177_MOESM8_ESM.pdf]

TAG

FFA

2119

2119+TadA

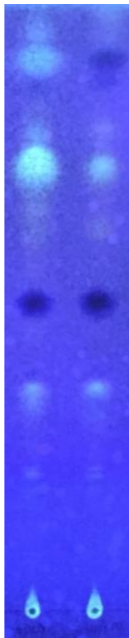

Supplement: Supplementary file 9 — Additional file 9: Figure S9. Overexpression of an LD-associated protein-encoding gene TadA decreased the TAG titer in strain 2119. Cells were cultured in ZYP-5052 auto-induction medium at 37°C with shaking at 200 rpm for 48 hr. Lipids extracted from 5 mg dried cells were loaded on TLC plate. Strain 2119, E.coli BL21(DE3) harboring pCDFDuet::tDGAT/RoPAP::RoFadD1/RoTetR2. [file 13068_2018_1177_MOESM9_ESM.pdf]
