# Supplementary material for: Stepwise metabolic engineering of Escherichia coli to produce triacylglycerol rich in medium-chain fatty acids
Source: Biotechnol Biofuels. 2018 Jun 25;11:177. doi: 10.1186/s13068-018-1177-x (PMC6016142; doi:10.1186/s13068-018-1177-x)
Supplement: Supplementary file 10 — Additional file 10: Table S1. 16 media were designed based on the ZYP-5052 auto-induction medium for improving the accumulation of TAGs rich in MCFAs. [file 13068_2018_1177_MOESM10_ESM.docx]

**Table S1.** 16 media were designed based on the auto-induction medium ZYP-5052 for improving the accumulation of TAG rich in medium-chain fatty acid (MCFA).

|  | N-Z-amine | glycerol | MgSO_4_ | (NH_4_)_2_SO_4_ | Aspartic acid + Serine | Yeast extract | Na_2_HPO_4_ | KH_2_PO_4_ | Glucose | Lactose | Trace metals^a^ |
| --- | --- | --- | --- | --- | --- | --- | --- | --- | --- | --- | --- |
|  | (%) | (%) | (mM) | (mM) | (%) | (%) | (mM) | (mM) | (%) | (%) |  |
| ZYP-5052 | 1 | 0.5 | 2 | 25 | 0 | 0.5 | 50 | 50 | 0.05 | 0.2 | 0.2× |
| 1 | 1 | 2 | 0.2 | 5 | 0.1 | - | - | - | - | - | - |
| 2 | 1 | 3 | 1 | 10 | 0.2 | - | - | - | - | - | - |
| 3 | 1 | 4 | 2 | 20 | 0.3 | - | - | - | - | - | - |
| 4 | 1 | 5 | 3 | 25 | 0.5 | - | - | - | - | - | - |
| 5 | 2 | 2 | 2 | 10 | 0.5 | - | - | - | - | - | - |
| 6 | 2 | 3 | 3 | 5 | 0.3 | - | - | - | - | - | - |
| 7 | 2 | 4 | 0.2 | 25 | 0.2 | - | - | - | - | - | - |
| 8 | 2 | 5 | 1 | 20 | 0.1 | - | - | - | - | - | - |
| 9 | 3 | 2 | 3 | 20 | 0.2 | - | - | - | - | - | - |
| 10 | 3 | 3 | 2 | 25 | 0.1 | - | - | - | - | - | - |
| 11 | 3 | 4 | 1 | 5 | 0.5 | - | - | - | - | - | - |
| 12 | 3 | 5 | 0.2 | 10 | 0.3 | - | - | - | - | - | - |
| 13 | 4 | 2 | 1 | 25 | 0.3 | - | - | - | - | - | - |
| 14 | 4 | 3 | 0.2 | 20 | 0.5 | - | - | - | - | - | - |
| 15 | 4 | 4 | 3 | 10 | 0.1 | - | - | - | - | - | - |
| 16 | 4 | 5 | 2 | 5 | 0.2 | - | - | - | - | - | - |

^a^A stock solution of 0.1 M FeCl_3_ was dissolved in a 100-fold dilution of concentrated HCl (final concentration 0.12 M HCl). This solution was combined with autoclaved stock solutions of other metals to make a 1000× trace metal mixture containing 50 mM FeCl_3_, 20 mM CaCl_2_, 10 mM each of MnCl_2_ and ZnSO_4_, and 2 mM each of CoCl_2_, CuCl_2_, NiCl_2_, Na_2_MoO_4_, Na_2_SeO_3_, and H_3_BO_3_ in 60 mM HCl. These solutions were stored at room temperature. Upon prolonged storage, small amounts of precipitate formed in the mixture.
