# Supplementary material for: Stepwise metabolic engineering of Escherichia coli to produce triacylglycerol rich in medium-chain fatty acids
Source: Biotechnol Biofuels. 2018 Jun 25;11:177. doi: 10.1186/s13068-018-1177-x (PMC6016142; doi:10.1186/s13068-018-1177-x)
Supplement: Supplementary file 11 — Additional file 11: Table S2. Strains and plasmids used in this study. [file 13068_2018_1177_MOESM11_ESM.docx]

**`Table S2.** Strains and plasmids used in this study.

| **Plasmid/Strain** | **Description** | **Source** |
| --- | --- | --- |
| **Plasmid** |  |  |
| pET28a(+) | T7 promoter, Kan^R^ ori pBR322 | Novagen |
| pCDFDuet-1 | Two T7 promoters, two MCS, Str^R^ ori | Novagen |
| pACYCDuet-1 | Two T7 promoters, two MCS, Amp^R^ ori P15A | Novagen |
| pCDFDuet:*:RoPAP* | pCDFDuet-1 containing *RoPAP* from *R. opacus* PD630 | This study |
| pCDFDuet:*:RjPAP* | pCDFDuet-1 containing *RjPAP* from *R. jostii* RHA1 | This study |
| pCDFDuet::*atfA*::*RoPAP* | pCDFDuet-1 containing a*tfA* from *A.r* *baylyi ADP1* and *RoPAP* from *R. opacus* PD630 | This study |
| pCDFDuet::*atf1*::*RoPAP* | pCDFDuet-1 containing *atf1* from *R. opacus* PD630 and *RoPAP* | This study |
| pCDFDuet::*atf2*::*RoPAP* | pCDFDuet-1 containing *atf2* from *R. opacus* PD630 and *RoPAP* | This study |
| pCDFDuet::*atf8*::*RoPAP* | pCDFDuet-1 containing *atf8* from *R. jostii* RHA1 and *RoPAP* | This study |
| pCDFDuet::*atfA_co*::*RoPAP* | pCDFDuet-1 containing *atfA* with codon optimization and *RoPAP* | This study |
| pCDFDuet::*tDGAT*::*RoPAP* | pCDFDuet-1 containing *tDGAT* from *T. curvata* and *RoPAP* | This study |
| pCDFDuet::*atfA*::*RjPAP* | pCDFDuet-1 containing *atfA* from *A.baylyi ADP1* and *RjPAP* from *R. jostii* RHA1 | This study |
| pCDFDuet::*tDGAT/RoPAP*::*RoFadD1* | pCDFDuet-1 containing *tDGAT*, *RoPAP* and *RofadD1* from *R. opacus* PD630 | This study |
| pCDFDuet::*tDGAT/RoPAP*::*RoFadD2* | pCDFDuet-1 containing *tDGAT*, *RoPAP* and *RofadD2* from *R. opacus* PD630 | This study |
| pET:*:fadR* | pET28a(+) containing *fadR* from *E. coli* MG1655 | [28] |
| pET:*:RoTetR1* | pET28a(+) containing *RoTetR1* from *R. opacus* PD630 | [28] |
| pET:*:RoTetR2* | pET28a(+) containing *RoTetR2* from *R. opacus* PD630 | [28] |
| pET:*:RoTetR3* | pET28a(+) containing *RoTetR3* from *R. opacus* PD630 | [28] |
| pCDFDuet::*tDGAT*/*RoPAP*::*RoFadD1*/*RoTetR2* | pCDFDuet-1 containing *tDGAT*, *RoPAP*, *RofadD1* and *RoTetR2* | This study |
| pACYCDuet::*CnFatB3* | pACYCDuet-1 containing *CnFatB3* | [28] |
| pACYCDuet::*CcFatB1* | pACYCDuet-1 containing *CcFatB1* | [28] |
| pACYCDuet::*CpFatB2* | pACYCDuet-1 containing *CpFatB2* | [28] |
| pACYCDuet::*ChFatB* | pACYCDuet-1 containing *ChFatB* | This study |
| pACYCDuet::*AcTesA’* | pACYCDuet-1 containing *AcTesA’* | This study |
| pACYCDuet::*RcFatA* | pACYCDuet-1 containing *RcFatA* | This study |
| pACYCDuet::*RcFatB* | pACYCDuet-1 containing *RcFatB* | This study |
| pCDFDuet::*tDGAT*/*RoPAP*::*RoFadD1*/*RoTetR2/RcFatB* | pCDFDuet-1 containing *tDGAT*, *RoPAP*, *RofadD1*, *RoTetR2* and *RcFatB* | This study |
| pET::*TadA* | pET28a(+) containing *TadA* from *R. opacus* PD630 | This study |
| **Strain** |  |  |
| *E. coli* DH5α | F^-^,φ80d*lacZ* ΔM15, Δ(*lac*ZYA -*arg*F )U169, *deo*R, *rec*A1, *end*A1, *hsd*R17 (rK^-^, mK^+^), *pho*A, *sup*E44, *λ^-^*, *thi*-1, *gyr*A96, *rel*A | Invitrogen |
| *E. coli* BL21(DE3) | *F- ompT gal dcm lon hsdSB(rB-mB-)λ(DE3)* | Invitrogen |
| 2119 | *E. coli* BL21(DE3) carrying pCDFDuet:: *tDGAT*/*RoPAP*:: *RoFadD1*/*RoTetR2* | This study |
