# Supplementary material for: Stepwise metabolic engineering of Escherichia coli to produce triacylglycerol rich in medium-chain fatty acids
Source: Biotechnol Biofuels. 2018 Jun 25;11:177. doi: 10.1186/s13068-018-1177-x (PMC6016142; doi:10.1186/s13068-018-1177-x)
Supplement: Supplementary file 12 — Additional file 12: Table S3. Primers used in this study. [file 13068_2018_1177_MOESM12_ESM.docx]

**Table S3.** Primers used in this study.

| **Primer name** | **Sequence（5’-3’）** |
| --- | --- |
| **For vector construction** |  |
| AtfA_F | GGAATTCGATGCGCCCATTACATCCG |
| AtfA_R | GAATGCGGCCGCATTGGCTGTTTTAATATC |
| AtfA_co_F | GGAATTCGATGCGCCCATTACATCCG |
| AtfA_co_R | GAATGCGGCCGCATTGGCTGTTTTAATATC |
| Atf1_F | CGGAATTCATGACCGACGTGAGCACGACG |
| Atf1_R | CCGCTCGAGTCGAGGCCACGACCACCCTCG |
| Atf2_F | CGGGATCCGATGCCGGTTACCGATTCG |
| Atf2_R | CCCAAGCTTGAGCAATGCCGCCTCGAG |
| Atf8_F | CGGAATTCGATGCCGCTGCCGATGTCTCCG |
| Atf8_R | GTTTAGCGGCCGCTTAGATACCAACAGCACGTTC |
| RoPAP_F | GGCATATGATGCCCCACACCTCCATC |
| RoPAP_R | GGAAGCTTAGGCCTCCCACTCGGT |
| RjPAP_F | GGCATATGATGCCCCACACCTCCATCGCCA |
| RjPAP_R | GGAAGCTTGCCTCCCACTCGGTCG |
| RoTetR2_F | ATTCCATATGATGGACCGCAACCGGA |
| RoTetR2_R | GCTCAATTGAGCGACTTGCGGAAAGCGTC |
| CnFatB3_F | GAAGATCTCATGGTTGCTTCTGTTGCTGC |
| CnFatB3_R | CCGCTCGAGTTTAGATTCGGTCGGGTGCAG |
| CcFatB1_F | GAAGATCTCATGGCTACCACCTCTCTGGC |
| CcFatB1_R | CCGCTCGAGAACAGAAGATTCAGCCGGGAT |
| CpFatB2_F | GAAGATCTCATGGTTGCTGCTGCTGCCAG |
| CpFatB2_R | CCGCTCGAGAGAGATAGAGTTACCG |
| ChFatB_F | GAAGATCTcATGGTTGCAGCAGCTGCTTC |
| ChFatB_R | CCGCTCGAGGGAAACAGAATTAC |
| AcTesA’_F | GAAGATCTCATGGCAAAAACCATTCTGAT |
| AcTesA’_R | CCGCTCGAGTAAAGCGCCTTTAATATATGGGT |
| RcFatA_F | GAAGATCTCATGCTGAAAGTTCCGTGCTGCAAC |
| RcFatA_R | CTCGAGACGAGCAGATTTTTTACGCCATTC |
| RcFatB_F | GAAGATCTCATGGTTGCTACCGCTGCTGCTGCTAC |
| RcFatB_R | CTCGAGAGCAGATTCAACCGGGATCTGACCCA |
| TadA_F | CGGAATTCATGACCGACCAGAAAACCATC |
| TadA_R | CCGCTCGAGAGCTTTTTTAGCCGGAGC |
| RoFadD1_F | TGCAAGAAACCGCTCTGAAC |
| RoFadD1_R | CAACCAGAACCTGCATACGG |
| RoFadD2_F | CGGGATCCATGCTCAACCTTTCCGTCCTTC |
| RoFadD2_R | CCCAAGCTTGAGTGCGGCCGGGGCGCGGCCGAGTT |
| tDGAT_F | CGGAATTCGATGCGTCAGTTAACAGCAGTTGATG |
| tDGAT_R | CTTTTGCGGCCGCTCAACCATCAATCAGACCACGCAGTTCACCCAG |
| 2119MCS1-F | CGGAATTCGATGCGTCAGTTAACAGCAGTTGATG |
| 2119MCS1-R | GTTTAGCGGCCGCTTAGCCTCCCACTCGGTCGAC |
| 2119MCS1-MR (with RBS) | CTCCTTTCAACCATCAATCAGACCACG |
| 2119MCS1-MF (with RBS) | GATGGTTGAAAGGAGATGCCCCACACCTCTGCCGCTC |
| 2119MCS2-F | TAAGTATAAGAAGGAGATATACATATGATGAACTCTCGTGACGCCGC |
| 2119MCS2-R | GCAGCGGTTTCTTTACCAGACTCGAGCGACTTGCGGAAAGCGTCCAG |
| 2119MCS2-MR (with RBS) | CGGTCCATCTCCTTTCAGCGACCCGAATACAGCTC |
| 2119MCS2-MF (with RBS) | GCAGCGGTTTCTTTACCAGACTCGAGCGACTTGCGGAAAGCGTCCAG |
